# Symptomatic Giant Virchow-Robin Spaces: A Rare Cause of Spastic Quadriparesis in 43-Year-old Ethiopian Patient: A Case Report

**DOI:** 10.4314/ejhs.v30i5.24

**Published:** 2020-09

**Authors:** Biniyam Ayele, Guta Zenebe, Abenet Mengesha, Yegeta Teshale

**Affiliations:** 1 Department of Neurology College of Health Sciences Addis Ababa University

**Keywords:** Dilated Virchow-Robin spaces, Magnetic resonance imaging, Spastic quadriparesis

## Abstract

**Background:**

Virchow-Robin Spaces (VRS) are perivascular spaces that surround small arteries and arterioles. These normal anatomical structures are thought to be involved in the drainage of interstitial fluid and also to play an immunomodulatory role by hosting macrophages. Rarely, it becomes giant and symptomatic resulting in mass effect on adjacent neuronal structures and ventricular system causing different neurological disorders.

**Case Presentation:**

We report a 43-year-old, Ethiopian woman who presented with progressive weakness of all her extremity over the period of seven years. She had associated speech difficulty, visual blurring and pseudo-bulbar affect. Neurologic examination revealed spastic quadriparesis with increased deep tendon reflexes and up going plantar bilaterally. She had horizontal nystagmus, dysarthria and reduced bilateral visual acuity, otherwise normal cognition and cranial nerves examination. Brain MRI showed T1 hypointense, T2 hyperintense and non-enhancing multiple cystic lesions of different size, mainly in bilateral basal ganglia area with mass effect on adjacent internal capsule and lateral ventricles. Considering her clinical presentation and typical radiological features, diagnosis of symptomatic dilated Virchow-Robin spaces was made, and the patient was treated symptomatically.

**Conclusion:**

Commonly, dilation of Virchow-Robin spaces are not symptomatic, but giant Virchow-Robin spaces, as in our patient may result in spastic quadriparesis, causing great disability on the patient. Thus, we recommend considering symptomatic Virchow-Robin spaces as a potential differential diagnosis of progressive quadriparesis, as early neurosurgical intervention may reduce the neurological complications, such as spastic quadriparesis.

## Introduction

Virchow-Robin Spaces (VRS) are potential spaces that surround the walls of cerebral vessels as they course from the subarachnoid space and penetrating the brain parenchyma. These normal anatomic existences have been implicated as a potential route for many pathogens and immunological factors to easily reach cerebral cortex, bypass the tight Blood Brain Barriers (1). VRS dilatation is said to be clinically significant when the size of the cyst become >3–5mm (1). The exact cause behind the dilatation of VRS has not been understood till now, but impaired permeability of the walls of the arterial vessels they surround is said to be one presumed underlying cause (2). Diagnosis of dilated VRS can be made with high certainty, by using high resolution brain MRI, and most importantly, by recognizing typical brain areas often affected by dilated VRS (3).

## Case Presentation

**Figure 1:**
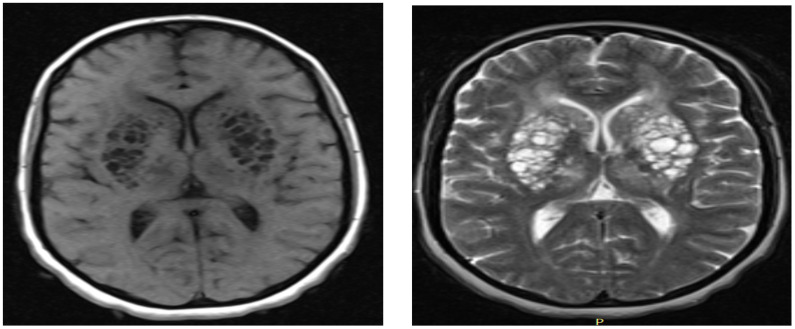
**a**) Axial T1 weighted MRI showing bilateral multiple hyporintense cystic lesions in basal ganglia region having mass effect on adjacent ventricles by making it like a slit; **b**) Axial T2 weighted MRI showing bilateral multiple hyporintense cystic lesions in basal ganglia region having mass effect on adjacent ventricles making it slit

**Figure 2:**
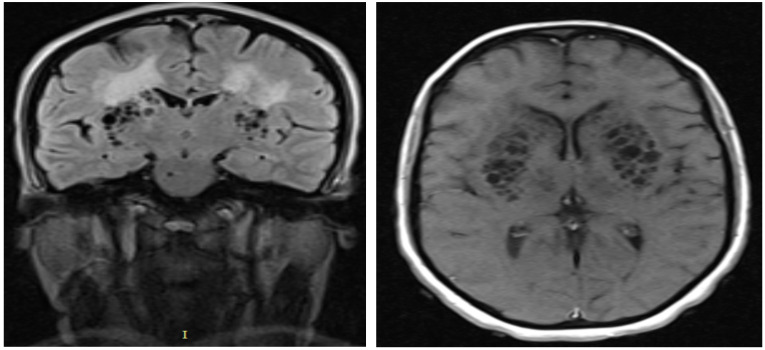
**a)** Coronal FLAIR MRI showing bilateral multiple cystic lesions in basal ganglia region having CSF-like content with increased signal intensity of the surrounding brain parenchyma; **b**) Axial T1 weighted MRI after gadolinium injection, showing bilateral non-enhancing multiple hyporintense cystic lesions in basal ganglia region

**Figure 3:**
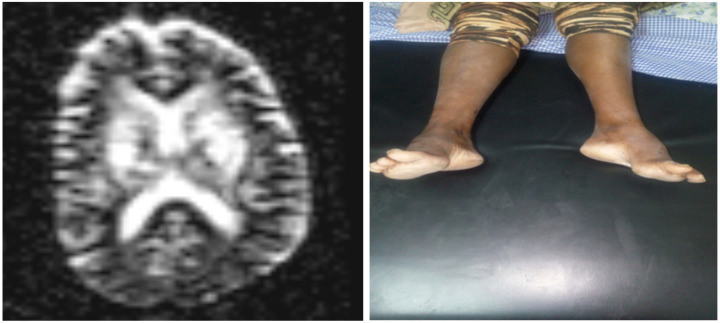
**a)** Diffusion-weighted image (DWI) of apparent diffusion coefficient (ADC) map shows no restricted diffusion in the affected areas of the brain; **b)** Bilateral pes-cavus, likely due to chronic lower limbs weakness

A 43-year-old woman presented to Yehuleshet Specialty Clinic with history of progressive weakness of all her extremities over the past seven years. She was apparently healthy before the onset of the weakness. The weakness started from her left leg and subsequently involved her right leg and since the past two months weakness involved both her upper extremities forcing her to be dependent on her family care for day-to-day activities, including feeding and dressing. Since the past two months, she became wheel chair bounded. She had speech difficulty, blurring of vision, headache, difficulty of swallowing, and since 3 weeks, she developed urinary and fecal incontinence. She had history of sudden inappropriate laughter episodes. Otherwise, she had no history of head trauma or chronic medical illness. She was educated up to grade 10 and was working on her private business before the onset of the illness. She had 3 siblings and both of her parents were alive and healthy. Neurological examination showed bilateral reduced visual acuity, horizontal nystagmus, dysarthria, and spastic quadriparesis with increased deep tendon reflexes and upgoing plantars bilaterally. Cognitive assessment and cranial nerves examination were normal. She had bilateral pes-cavus (Figure 3b). Liver and renal functions and lipid profiles are normal. A total blood count was normal. Serological tests for HIV, HCV, HBV, and syphilis were negative. Chest X-ray, Echocardiography, stool examination, urine analysis, and Erythrocyte sedimentation rate (ESR) were unremarkable. Brain MRI showed multiple, bilateral, and symmetrical T1 hypointense, T2 hyperintense, and rounded lesions (Figure 1a, 1b). The cystic lesions shows CSF-like content with increased signal intensity of the surrounding brain parenchyma on FLAIR sequences (Figure 2a). The lesions are non-enhancing to contrast material (Figure 2b) and predominantly observed in basal ganglia area causing mass effect on adjacent descending motor fibers and lateral ventricles. A single lesion was also seen in right pontomesencephalic region (Figure 2a). Diffusion-weighted image apparent diffusion coefficient map shows no restricted diffusion in the affected part of the brain (Figure 3a). Cervical, lumbosacral MRI and nerve conduction study of lower extremity were normal. Neurosurgical team was consulted for possible surgical intervention, but they deferred surgical management and advised to optimize conservative management strategy. Currently, the patient has been managed in the following ways: twice weekly physiotherapy to improve her spasticity and to prevent contracture, amitriptyline 25mg daily was started for pseudobalbar affect and associated neuropathic pain, and home care. Home care focus on optimizing feeding and bowel/bladder care to avoid infections and, advised on importance of follow-up evaluation every three months.

**Ethics approval and consent to participate**: The authors' institution does not require ethical approval for publication of a single case report. Written informed consent was obtained from the patient. Written informed consent for publication of clinical details and images was obtained from the patient.

## Discussion

Rarely, dilated VRS became giant to attain large size and become multiple in numbers to become symptomatic causing significant mass effect on adjacent neural structures and ventricular systems (1). In our patient, the giant and multiple symptomatic VRS caused mass effect on basal ganglia area and adjacent internal capsule only to result in progressive spastic quadriparesis with pseudo-bulbar affects - a clinical feature never been reported previously with dilated VRS. The single cyst in right pontomesencephalic area is believed to be responsible for the horizontal nystagmus and dysarthria observed in our patient. Heier LA, et al, reported features that strongly predict giant VRS such as advanced age, arterial hypertension, dementia, and incidental white matter lesions (4). In contrary to this report, our patient was relatively young and has no hypertension, but had white matter hyperintensity on MRI (Figure 3a). Dilated VRS is classified as type I, type II, and type III based on the location of the dilated CRS (4). Our patient is categorized under mixed type I and III dilated VRS (Figure 1a, 1b, 2a, 2b, 3a).

Typical MRI signal intensities and locations allow distinguishing dilated VRS from other potential mimickers such as (a) chronic lacunar infarction: lacunar infarctions are generally often large in size > 5mm, wedged shaped, generally not symmetrical and have hyperintense on FLAIR sequence, in addition to absences of conventional risk factors speaks against, (b) mucopolysaccharidoses: patients often have brain atrophy, mental retardation and abnormalities of the white matter. However, normal childhood motor development, cognition and absent macrocephaly, normal cervical and sellar region speak against diagnosis of mucopolysaccharidoses less likely, (c) cystic periventricular leukomalacia (CPL): often have brain MRI features of: increased signal intensity on T2 and FLAIR sequences with thinning of corpus callosum. The patient age and hypointensity on FLAIR sequence speaks against CPL. Finally, negative HIV serology status makes diagnosis of cryptococcosis in our patient less likely.

To our knowledge, this is the first case of symptomatic dilated VRS to be reported from Ethiopia. Thus, we recommend considering symptomatic dilated VRS as a potential differential diagnosis for a patient who present with clinical features of progressive spastic quadriparesis and suggestive brain MRI findings.
